# Near‐infrared reflectance spectroscopy phenomic prediction can perform similarly to genomic prediction of maize agronomic traits across environments

**DOI:** 10.1002/tpg2.20454

**Published:** 2024-05-07

**Authors:** Aaron J. DeSalvio, Alper Adak, Seth C. Murray, Diego Jarquín, Noah D. Winans, Daniel Crozier, William L. Rooney

**Affiliations:** ^1^ Interdisciplinary Graduate Program in Genetics and Genomics (Department of Biochemistry and Biophysics) Texas A&M University College Station Texas USA; ^2^ Department of Soil and Crop Sciences Texas A&M University College Station Texas USA; ^3^ Department of Agronomy University of Florida Gainesville Florida USA

## Abstract

For nearly two decades, genomic prediction and selection have supported efforts to increase genetic gains in plant and animal improvement programs. However, novel phenomic strategies for predicting complex traits in maize have recently proven beneficial when integrated into across‐environment sparse genomic prediction models. One phenomic data modality is whole grain near‐infrared spectroscopy (NIRS), which records reflectance values of biological samples (e.g., maize kernels) based on chemical composition. Predictions of hybrid maize grain yield (GY) and 500‐kernel weight (KW) across 2 years (2011–2012) and two management conditions (water‐stressed and well‐watered) were conducted using combinations of reflectance data obtained from high‐throughput, F_2_ whole‐kernel scans and genomic data obtained from genotyping‐by‐sequencing within four different cross‐validation (CV) schemes (CV2, CV1, CV0, and CV00). When predicting the performance of untested genotypes in characterized (CV1) environments, genomic data were better than phenomic data for GY (0.689 ± 0.024—genomic vs. 0.612 ± 0.045—phenomic), but phenomic data were better than genomic data for KW (0.535 ± 0.034—genomic vs. 0.617 ± 0.145—phenomic). Multi‐kernel models (combinations of phenomic and genomic relationship matrices) did not surpass single‐kernel models for GY prediction in CV1 or CV00 (prediction of untested genotypes in uncharacterized environments); however, these models did outperform the single‐kernel models for prediction of KW in these same CVs. Lasso regression applied to the NIRS data set selected a subset of 216 NIRS bands that achieved comparable prediction abilities to the full phenomic data set of 3112 bands predicting GY and KW under CV1 and CV00.

## Core Ideas

- Near‐infrared spectroscopy (NIRS) phenomic and genomic prediction had similar accuracies for grain yield and 500‐kernel weight.
- Models with only genomic/phenomic data often outperformed multi‐kernel models.
- Lasso regression removed correlated NIRS bands with limited reduction in prediction ability.

**Abbreviations**
CV: cross‐validation
GBS: genotyping‐by‐sequencing
GY: grain yield
KW: 500‐kernel weight
NIRS: near‐infrared spectroscopy
PP: phenomic prediction
UAS: unoccupied aerial system
WS: water‐stressed
WW: well‐watered

## 1 INTRODUCTION

Near‐infrared reflectance (NIR) spectroscopy (NIRS) is an active remote sensing approach that leverages techniques in spectroscopy, analytical chemistry, and mathematics (specifically chemometrics) (Pasquini, 2018). NIRS functions by barraging a sample with electromagnetic radiation at wavelengths between 750 and 2500 nm, producing absorption or reflectance values of any compound containing C–H, N–H, S–H, or O–H chemical bonds (Pasquini, 2003, 2018). NIRS measurements are generally recorded in laboratory settings using dried tissue or grain (Ferrio et al., 2004), although portable spectrometers can provide active or passive, non‐destructive measurements of plant tissue in the field. NIR spectrometers assess each sample as a single measurement at each discrete band and differ from hyperspectral NIR imaging, which creates a “hyperspectral data cube” with spatial information but generally measures fewer discrete bands. Hernandez et al. (2015) demonstrated the ability to predict grain yield (GY) using canopy NIRS measurements collected at anthesis and grain fill as a proxy trait for GY in a diverse wheat panel. Recently, some small plot combines and forage harvesters have been equipped with NIRS to minimize labor and bias of subsampling grain or biomass for composition (Polytec GmbH, 2022). Given the existing prevalence of NIRS in plant science and breeding programs for kernel composition and quality testing, many researchers are already equipped to incorporate NIRS as a method for phenomic prediction (PP) using reflectance values as phenomic markers instead of genomic markers, in turn facilitating PP and potentially phenomic selection (PS). In the first major NIRS‐based PP study, Rincent et al. (2018) demonstrated the ability of NIR spectra to enable prediction accuracy of complex traits on par with marker‐based prediction, despite subjecting the models to environments distinct from the one in which NIRS data were recorded. The study employed covariance matrices based on Yamada et al. (1988) to predict wheat yield in a separate environment, highlighting the role NIRS‐based relationship matrices can play in demarcating genetic relationships between lines. NIR spectra have been used previously to estimate complex plant compositional traits and then to predict GY based on the relationships that exist between GY and composition. Spectra can also be implemented in combination with molecular markers to bolster genomic prediction accuracy or coupled with a more commonly recognized genomic relationship (kinship) matrix (Robert, Brault et al., 2022). Phenomic prediction was proposed on the basis that phenomic markers, such as those afforded by NIRS, capture genetic relatedness, enabling across‐environment predictions from spectra obtained in different environments. This concept, demonstrated in Rincent et al. (2018), produced predictions with higher accuracy at times than genomic prediction (Robert, Brault et al., 2022). Weiß et al. (2022) reported more stable prediction ability of NIRS phenomic versus genomic prediction when predicting dry grain matter content, GY, and phosphorus concentration across diverse breeding material. Weiß et al. (2022) also found genomic prediction to be less tolerant to genetic divergence between training and test data sets. Robert, Auzanneau et al. (2022) demonstrated the combined predictive ability of genotypic and NIRS data and emphasized the impact of environment on predictive ability as captured by NIRS using 2 years of winter bread wheat candidate breeding lines. In a large panel of soybean recombinant inbred lines, Zhu et al. (2021a) found NIRS‐based PP ability to be comparable, and in some cases greater than, genomic prediction ability within and between segregating families. Zhu et al. (2021a) also found that NIRS data sets from distinct environments can be substituted for data sets from other environments and applied successfully in PP. This is relevant for predicting how genotypes will perform in untested environments, paving the way for translatable applications of PP and eventual selection.

In this study, building on NIRS data from Lane et al. (2020) and genomic data from Farfan et al. (2015), phenomic (NIRS) and genomic data of maize lines evaluated as hybrids on a common tester were used as predictors either separately or together to predict GY and 500‐kernel weight (KW) using a novel analysis approach. Notably, we accessed genomic data not presented in Lane et al. (2020) to directly compare genomic and PP in addition to further dissecting the phenomic data set using lasso regression to uncover autocorrelated NIRS bands. To assess prediction accuracy under relevant plant breeding scenarios, the performance of tested and untested maize hybrids was predicted in observed and unobserved environments across water‐stressed (WS) and well‐watered growth conditions over 2 years (four environments total) under four distinct cross‐validation (CV) schemes. In summary, this research sought to (i) develop combined genomic and NIRS models capable of predicting traits such as GY and KW across environments; (ii) assess the utility of including NIR spectra in predictive models alone and alongside genomic data; (iii) explore the predictive ability of a subset of NIRS bands nominated/selected by lasso variable importance scores for predicting GY and KW; and (iv) examine the performance of multi‐kernel genomic and PP models versus single‐kernel models.

## 2 MATERIALS AND METHODS

### 2.1 Experimental design

Field trials were conducted at the Texas A&M AgriLife Experiment Station in Burleson County, TX, during the summers of 2011 and 2012, as described by Farfan et al. (2015). Briefly, a subset of 346 maize lines from the 302‐line USDA Goodman maize association panel (Flint‐Garcia et al., 2005) and the 300‐line diverse southern subtropical Williams/Warburton panel (Warburton et al., 2013) were testcrossed to two variants of Tx714 (Betrán et al., 2004). These two variants of Tx714, isogenic lines with lipoxygenase 4 and 5 knockouts, were not significantly different for any measured trait and were therefore combined throughout analysis. Testcross hybrids were grown in randomized complete block designs with two replications in each year/management combination, consisting of well‐watered(WW) and WS management conditions in 2011 and 2012. These will hereafter be denoted as CS11_WS, CS11_WW, CS12_WS, and CS12_WW, indicating four different growing environments that contained 196, 263, 310, and 213 hybrids, respectively (Figure S1). A common set of 146 hybrids formed the basis of the train/test split that enabled sparse genomic and PP.

### 2.2 Agronomic traits

GY and 500‐kernel weight (KW) were recorded on a plot basis, and values are reported in t/ha and mass in grams of 500 kernels, respectively.

**Core Ideas**

- Near‐infrared spectroscopy (NIRS) phenomic and genomic prediction had similar accuracies for grain yield and 500‐kernel weight.
- Models with only genomic/phenomic data often outperformed multi‐kernel models.
- Lasso regression removed correlated NIRS bands with limited reduction in prediction ability.

### 2.3 Phenomic data: Near‐infrared spectroscopy

High‐throughput, whole‐kernel NIRS scans were obtained with a Thermo Scientific Antaris II Fourier transformed interferometer (Thermo Fisher Scientific) using calibrations reported in Meng et al. (2015) and Christman (2017). NIRS data from Lane et al. (2020) from grain harvested from the testcross hybrids developed by Farfan et al. (2015) were used. In brief, whole grain samples of 196 (CS11_WS), 269 (CS11_WW), 310 (CS12_WS), and 213 (CS12_WW) hybrids were scanned in a rotating cup using the interferometer. Scanning and spectral processing methodologies are described in Meng et al. (2015), and initial analyses and quality control parameters are discussed in Lane et al. (2020). As a result, reflectance values corresponding to 3112 NIRS bands were extracted from each sample. A brief overview of the scanning methodology is as follows: (a) whole grain samples in ∼175 g batches were analyzed in triplicate with 128 scans per sample; (b) with the scanner set to record diffuse reflectance, scans were performed over the instrument's integrating sphere module; (c) all spectra were recorded at ambient temperature and were calculated at 4 cm^−1^ resolution, thus encompassing 4000–10,000 cm^−1^; (d) preprocessing techniques were employed such as multiplicative signal correction, standard normal variate, data treatment with first and second derivative transformations, and Savitzky‐Golay filtering was performed using Thermo Scientific's TQ Analyst Pro Edition Software (Meng et al., 2015).

Using the *caret* package in R (Kuhn, 2008), the lasso algorithm was run with fivefold three‐replication CV using the maximum available hybrids in each environment to determine the most important NIRS bands (cm^−1^) associated with GY and kernel composition traits. Model performance was assessed using *R* ^2^ (DeSalvio, 2023). The lasso algorithm nominated important NIRS bands linked to GY and KW in each environment. GY and KW were set as response variables within the *lasso* R package *train* function using fivefold CV, centering and scaling, and “*method = glmnet*.” Hyperparameter grid search settings were left at default, with alpha and lambda set to one and zero, respectively. Variable importance scores were extracted using the *varImp* function. By selecting bands with variable importance scores exceeding zero, lasso nominated 216 NIRS bands for GY and 131 bands for KW. No significant advantages in predictive ability were observed when constructing the matrix using the combined bands from GY and KW (347 total bands, 216 from GY and 131 from KW). As a result, phenomic, genomic, and combined prediction methods were evaluated after constructing the phenomic relationship matrix with the bands from GY only.

### 2.4 Genotypic data

Using the genotyping‐by‐sequencing (GBS) method outlined in Elshire et al. (2011), genome‐wide single‐nucleotide polymorphism (SNP) genotypic data were obtained for 213 lines from the Williams/Warburton panel from the USDA‐ARS Corn Host Plant Resistance Research Unit (Mississippi State University). Genotypic information was obtained from 133 of the 282 inbred lines comprising the USDA Goodman maize panel from Panzea (Zhao et al., 2006). These GBS data were later used for genomic and combined genomic/PP models. In this study, 346 total unique hybrids were planted, which consisted of 213 and 133 lines originating from the Williams/Warburton and USDA Goodman panels, respectively. The other 149 lines from the USDA Goodman maize panel are poorly adapted to Texas and did not generate sufficient testcross seed for yield trials and phenotyping.

Quality control of genomic data from an initial array of 61,410 polymorphic loci were performed as follows: (i) heterozygote calls were set as missing data; (ii) markers with >20% missing values were removed; and (iii) markers with <0.05 minor allele frequency were removed. After quality control, 41,456 markers (according to maize genome version AGPV2) were used to construct the genomic relationship matrix. A resulting set of 329 hybrids had genomic data of sufficient quality to be used for genomic and genomic/PP analyses (Figure S1).

### 2.5 Analysis of variance (ANOVA)

To estimate genetic and other experimental variance components, a multi‐environment ANOVA, Equation (1), was applied to the raw data for GY, KW, and each NIRS band in series (3112 bands total) to obtain the best linear unbiased predictor (BLUP) pedigree effects via the *lme4* package in R (Bates et al., 2015).

(1) Yjklmn=μ+Pedigreej+Envk+Env×Pedigreejk+Rangel+Rowm+Env×Repnk+εjklmn

*Y* is a vector of length 982 (the sum of the number of hybrids in all environments) denoting each trait value for each hybrid in one of the four environments for GY, KW, and the reflectance values for each of the 3112 NIRS bands for observations of the *j*th maize hybrid in the *k*th environment, *l*th range, *m*th row, and *n*th replication. Here, *μ* denotes the grand mean; Pedigree denotes the overall effect of the *j*th maize hybrid, where $\mathrm{Pedigre}e_{j}\;\overset{i.i.d.}{∼}\;N\left(0,\;\sigma_{j}^{2}\right)$; Env denotes the effect of the *k*th environment where $\mathrm{En}v_{k}\;\overset{i.i.d.}{∼}\;N\left(0,\;\sigma_{k}^{2}\right)$; Env×Pedigree denotes the effect of the *j*th hybrid within the *k*th environment where $\mathrm{Env}\times \mathrm{Pedigre}e_{jk}\;\overset{i.i.d.}{∼}\;N\left(0,\;\sigma_{jk}^{2}\right)$; Range denotes the effect of *l*th range where $\mathrm{Rang}e_{l}\;\overset{i.i.d.}{∼}\;N\left(0,\;\sigma_{l}^{2}\right)$; Row denotes the effect of the *m*th row where $\mathrm{Ro}w_{m}\;\overset{i.i.d.}{∼}\;N\left(0,\;\sigma_{m}^{2}\right)$; Env×Rep denotes the effect of the *n*th replication within the *k*th environment where Env×Repnk∼i.i.d.N(0,σnk2); and *ɛ* denotes the error term addressing the non‐explained variability with σ^2^_error_ as the associated variance component such that $\varepsilon_{jklmn}\overset{i.i.d.}{∼}N\left(0,\;\sigma_{\varepsilon }^{2}\right)$.

Repeatability was calculated using Equation (2).

(2) $$\mathrm{Repeatability}=\frac{\sigma_{\mathrm{Pedigre}e_{j}}^{2}}{\sigma_{j}^{2}+\;\frac{\sigma_{jk}^{2}}{k}+\;\frac{\sigma_{\varepsilon_{jklmn}}}{nk}\;}$$

Here, $\sigma_{j}^{2}$, $\sigma_{jk}^{2}$, and $\sigma_{\varepsilon_{jklm}}^{2}$ are the variance components of the overall effects of pedigree, the interaction between pedigree and environment, and the error term, respectively, *n* is the number of replicates, and *k* is the number of environments. Pedigree effects (BLUPs) for GY, KW, and each NIRS band are shown in the Supporting Information (DeSalvio, 2023).

### 2.6 Sparse prediction: ACROSS‐environment genomic and PPs

For sparse prediction across the four environments, the 146 maize hybrids common to all four environments were split approximately equally into fivefold, with fourfold used for training each model. The *BGLR* package was used in R to perform predictions of both GY and KW (Pérez & de Los Campos, 2014), considering genomic and/or phenomic covariates. Models were fit under a hierarchical Bayesian framework with 5000 Gibbs sampler iterations, 20% burn‐in, and the thinning parameter was set at 10 for a total of 400 iterations used to compute the posterior mode of the different parameters involved in the linear predictors. Environment effects were calculated using Bayesian ridge regression, and all other kernels were specified as Reproducing kernel Hilbert spaces (specified as “BRR” and “RKHS,” respectively, in the *BGLR* package documentation), which under this implementation corresponds to the conventional GBLUP approach.

Five genomic and phenomic best linear unbiased prediction (GBLUP/PBLUP) models, including main and interaction effects between genomic/phenomic markers and environments modeled with different covariance structures, were compared in this study (Table 1). The data preparation and matrix multiplication to obtain each kernel are described in the Supporting Information repository (DeSalvio, 2023) and in the following section. In the series of equations in Table 1, *Y* represents a 982×1 vector of BLUPs from Equation (1) for GY or KW across all four environments. Each element of $Y_{jkq}$ corresponding to the BLUP value for the *j*th hybrid in the *k*th environment (k=1,…,4). μ represents the grand mean, and $g_{j}$, $q_{j}$, and $qs_{j}$ denote the multi‐environment genomic, phenomic, and subset phenomic relationship matrices, respectively. $E_{k}$ represents the 982×4 environment incidence matrix, and $gE_{jk}$, $pE_{jk}$, and $psE_{jk}$ represent the interaction effect of environment with genomic, phenomic, and subset phenomic effects, respectively; $\varepsilon_{jkq}$ denotes the residual error addressing the non‐explained variability.

**TABLE 1:** Summary of prediction models for across‐environment predictions. M1 indicates genomic prediction; M2 indicates phenomic prediction; M3 indicates phenomic prediction in which the phenomic relationship matrix was constructed with a subset of near‐infrared spectroscopy (NIRS) bands nominated by lasso as important for predicting the trait of interest; M4 combines aspects of M1 and M2; and M5 combines aspects of M1 and M3.

Model	Formula	Description
M1	$\;Y_{jpq}=\mu +g_{j}+E_{k}+gE_{jk}+\varepsilon_{jk}$	Genomic
M2	$\;Y_{jpq}=\mu +q_{j}+E_{k}+qE_{jk}+\varepsilon_{jk}$	Phenomic
M3	$\;Y_{jpq}=\mu +q_{j}+E_{k}+qsE_{jk}+\varepsilon_{jk}$	Subset phenomic
M4	$\;Y_{jpq}=\mu +g_{j}+q_{j}+E_{k}+gE_{jk}+qE_{jk}+\varepsilon_{jk}$	Genomic + phenomic
M5	$\;Y_{jpq}=\mu +g_{j}+q_{j}+E_{k}+gE_{jk}+qsE_{jk}+\varepsilon_{jk}$	Genomic + subset phenomic

*Note*: The subset phenomic relationship matrix in M3 and M5 was constructed with 216 NIRS bands nominated as having high variable importance by lasso regression.

*Genomic data preparation*—Here, g symbolizes the multi‐environment additive genomic effect matrix, modeled via a linear combination between n additive markers and their corresponding effects based on the first method outlined by VanRaden (2008) using the *AGHmatrix* package in R (Amadeu et al., 2016). Because only one pollinator (Tx714) was used in the topcross, only an additive genomic relationship matrix was calculated. Preparation of the g matrix was as follows: (i) the complete 329×41,456 SNP matrix of all maize hybrids was used to create separate matrices of 196, 263, 310, and 213 rows, each with 41,456 columns, corresponding to SNPs for each hybrid grown within CS11_WS, CS11_WW, CS12_WS, and CS12_WW, respectively; (ii) each matrix was scaled and centered column‐wise; (iii) these matrices were then stacked to obtain a 982×41,456 SNP matrix denoted as $Z_{g}$; (iv) the multi‐environment g matrix was produced by matrix multiplication of $Z_{g}$ with its transpose and then divided by the number of SNPs (41,456), given by: $G\;=\frac{Z_{g}Z_{g}^{\prime }}{41,456}\;$, and g is 982×982. The genomic effects are considered outcomes from the normal distribution according to $g\overset{i.i.d.}{∼}N\left(0,\;G\sigma_{g}^{2}\right)$, $\sigma_{g}^{2}$ as the additive genetic variance. The interaction between the genotypes and the environments was modeled based on Jarquín et al. (2014) as the Hadamard product (⊙) between their corresponding covariance structures such that $Z_{E}$ represents the incidence matrix that connects phenotypic records with environments. Hence, the covariance structure of the interaction components is given by $\left[Z_{E}Z_{E}^{\prime }\right]\;⊙\;\left[\frac{Z_{g}Z_{g}^{\prime }}{41,456}\right]$, therefore $gE\;\overset{i.i.d.}{∼}\;N\left(0,\left[Z_{E}Z_{E}^{\prime }\right]\;⊙\;\left[\frac{Z_{g}Z_{g}^{\prime }}{41,456}\right]\sigma_{gE}^{2}\right)$, where the genotype × environment variance components are given by $\sigma_{gE}^{2}$.

*Phenomic data preparation: Entire data set*–Here, q symbolizes the multi‐environment phenomic effect matrix, modeled as a linear combination of 3112 NIRS bands and their effects within each environment. A similar data preparation procedure was implemented with NIRS data: (i) NIRS data corresponding to BLUPs of reflectance values for each hybrid in each environment were prepared as separate matrices of 196, 263, 310, and 213 rows with 3112 columns; (ii) each matrix was scaled and centered column‐wise; (iii) the matrices were stacked to obtain a 982×3112 matrix denoted as N; (iv) the multi‐environment phenomic relationship matrix was obtained by multiplying N by its transpose and dividing by the number of NIRS bands, given by: $q\;=\;\frac{NN^{\prime }}{3112}$, where q is 982×982. Each NIRS effect within q is assumed to be sampled from normal distribution with $N_{q}\overset{i.i.d.}{∼}N\left(0,\;\sigma_{q}^{2}\right)$, where the NIRS variance components are given by $\sigma_{q}^{2}$. The interaction between the environment kernel and the NIRS effect kernel was modeled with the covariance structure given by the Hadamard product of E and q: EE⊙q, therefore $qE\;\overset{i.i.d.}{∼}\;N\left(0,\left[Z_{E}Z_{E}^{\prime }\right]\;⊙\left[\frac{NN^{\prime }}{3112}\right]\;\sigma_{qE}^{2}\right)$. The NIRS × environment variance components are given by $\sigma_{qEE}^{2}$.

*Phenomic data preparation: Subset of NIRS bands determined by lasso*–Here, qs symbolizes the phenomic effects computed with the subset multi‐environment phenomic matrix. Identical data processing procedures were implemented above, except that in each instance, out of the 3112 initial bands, only 216 bands were used. These correspond to those with variable importance scores greater than zero as determined by the lasso function implemented via the *caret* package in R (Kuhn, 2008). To summarize, (i) NIRS data corresponding to BLUPs of reflectance values for each hybrid in each environment were again prepared as separate matrices of 196, 263, 310, and 213 rows, but now with only the 216 NIRS bands nominated by lasso; (ii) each matrix was scaled and centered column‐wise; (iii) the matrices were stacked to obtain a 982×216 matrix denoted as NS; (iv) the multi‐environment subset phenomic relationship matrix qs was obtained by multiplying NS by its transpose and dividing by the number of NIRS bands (now 216 vs. 3112 above), given by: $Qs\;=\;\frac{NSNS^{\prime }}{216}$, where Qs is 982×982. NIRS effects within qs are again assumed to be sampled from normal distributions with $qs∼N(0,\;Qs\sigma_{qs}^{2})$, and the variance components of the subset of NIRS bands are given by $\sigma_{qs}^{2}$. The interaction between the environment kernel and the subsetted NIRS effect kernel was modeled with the covariance structure given by the Hadamard product of E and ps: therefore, $psE\;∼\;N(0,\left[Z_{E}Z_{E}^{\prime }\right]\;⊙\left[\frac{NSNS^{\prime }}{216}\right]\;\sigma_{psE}^{2})$ or simply $qsE\;\overset{i.i.d.}{∼}\;N\left(0,\;\left[Z_{E}Z_{E}^{\prime }\right]\;⊙\;Qs\;\sigma_{psE}^{2}\right)$. The subset NIRS × environment variance components are given by $\sigma_{qsE}^{2}$.

### 2.7 Cross‐validation performance evaluation

The prediction performance of each model was assessed using a fivefold 20‐replication scheme. Four prediction scenarios were implemented based on the naming convention proposed by Jarquín et al. (2017), where CV2 represents prediction of tested genotypes in characterized environments, CV1 represents prediction of untested genotypes in characterized environments, CV0 represents prediction of tested genotypes in uncharacterized environments, and lastly, CV00 represents prediction of untested genotypes in uncharacterized environments. In CV1 and CV2, training data consisted of genotypes belonging to all four environments, so all environments were considered tested or characterized, whereas in CV0 and CV00, training data consisted of only three out of four environments, leaving one environment as uncharacterized or untested. CV schemes are visually described in Figure S2.

The 146 common hybrids grown in each of the four environments (Figure S1) were partitioned into fivefold, four of which were used to train each model. In CV1 and CV2, phenotypic observations belonging to hybrids from fourfold (116 out of 146, approximately 80%) in each of the four environments were kept, while all other values were set as missing values to be predicted by the model. In this way, the training data set consisted of 464 phenotypic records (116×4) while the evaluation set consisted of 518 records. The evaluation set was composed of 30 of 146 common hybrids not allotted to the training set in all four environments plus the remaining records from each of the four environments (30×4+398=518). The same 116 hybrids in all four environments were kept as training data, meaning that they were partitioned by genotype, not by pooling records from all four environments and randomly partitioning after pooling. The prediction accuracy of CV2 was evaluated as the correlation between actual GY or KW values of the 464 training records and predicted values of the same 464 training records. This simulated predicting tested hybrids in characterized environments since the training data set had phenotypic observations from all environments. The prediction accuracy of CV1 was evaluated as the correlation between actual values of hybrids not found in the training set (518 records) and predicted values of those records, which simulated prediction of untested hybrids in characterized environments since the training data set again had phenotypic observations from all environments (Figure S2).

In CV0 and CV00, the 146 hybrids common to all environments were again split into fivefold, with GY or KW values from fourfold (116 hybrids) being kept in only three of the four environments, meaning trait values from one of the environments (CS12_WW for the example in Figure S2) were set as missing values. In this way, the training data set is comprised strictly of records from four of fivefold (116 hybrids) from the 146 common hybrids in CS11_WS, CS11_WW, and CS12_WS (only three environments instead of four previously), and predictions for CS12_WW had to borrow information from the remaining three environments. In total, the training data size consisted of 348 phenotypic records (116×3) while the evaluation data set consisted of the remaining 634 records, amounting to 30 of 146 common hybrids in all four environments, the 146 records from the withheld environment, and the remaining 368 hybrids distributed throughout all four environments (116+(30×4)+368=634). All possible combinations of setting values from one of the four environments as missing were considered and are reported below.

The prediction accuracy of CV0 was again calculated as the correlation between actual and predicted values of the 116×4=464 common hybrids in all four environments, but the model was generated using records from only three environments. In this way, the model predicted tested hybrids in uncharacterized environments. For CV00, the prediction accuracy was calculated as the correlation between actual and predicted values of the remaining 518 hybrids, but in this scheme, none of the hybrids being predicted were in the training set. Here, the model predicted untested hybrids in uncharacterized environments (Figure S2).

### 2.8 Prediction ability

To quantify prediction ability of each CV/model/trait combination, a weighted average correlation was implemented according to Tiezzi et al. (2017). This calculation accounted for the unequal numbers of hybrids grown in each environment and ensured proper weighting of the results from environments with differing numbers of hybrids:

(3) $$r_{\varphi }=\frac{\sum_{i\;=\;1}^{I}\;\frac{r_{i}}{V\left(r_{i}\right)}}{\sum_{i\;=\;1}^{I}\;\frac{1}{V\left(r_{i}\right)}}$$

where $r_{i}$ is the Pearson's correlation between actual and predicted values belonging to the *i*th environment, $V\;\left(r_{i}\right)=\frac{1-r_{i}}{n_{i}-2}\;$ is the sampling variance, and $n_{i}\;$ is the number of hybrids in each environment.

## 3 RESULTS

### 3.1 Variance component analysis from multi‐environment ANOVA

**FIGURE 1:**
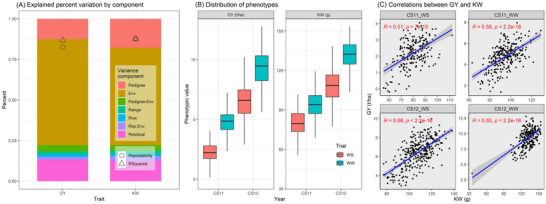
(A) Results are displayed for analysis of variance (ANOVA) of grain yield (GY) and 500‐kernel weight (KW) according to Equation (1), where Yjklmn=μ+Pedigreej+Envk+Env×Pedigreejk+Rangel+Rowm+Env×Repnk+εjklmn. Black circles denote repeatability, and white triangles indicate *R* ^2^ values. (B) Phenotypic data distributions are shown for each trait with red denoting water stress and blue denoting well‐watered conditions. Dashed lines indicate the means of each distribution. (C) Correlations between GY and KW are displayed across each environment. Statistically significant positive correlations were observed in all cases. CS, College Station; WS, water‐stressed; WW, well‐watered.

Results of ANOVA from Equation (1) indicated that for GY and KW, the environment component explained 65.2% and 59.9% of each trait, respectively (Figure 1A). Spatial variation (range, row, and the interaction between replication and environment) each individually explained little of the experimental variation, with the interaction between replication and environment displaying the largest effect at 7.00% for GY. *R* ^2^ and repeatability reached 0.868 and 0.826 for GY, respectively, and both metrics exceeded 0.874 for KW. Pedigree explained 19.7% and 12.3% of variation for GY and KW, while the interaction between pedigree and environment accounted for 4.04% and 3.57%, respectively, indicating relatively modest genotype × environment interaction. Distributions of pedigree BLUPs for GY and KW were stratified by environment, with the atypical drought conditions in CS11_WS visible in the low GY and KW values for this environment, whereas CS12_WW demonstrated the highest values for both traits (Figure 1B).

### 3.2 Variance component analysis for NIRS bands

**FIGURE 2:**
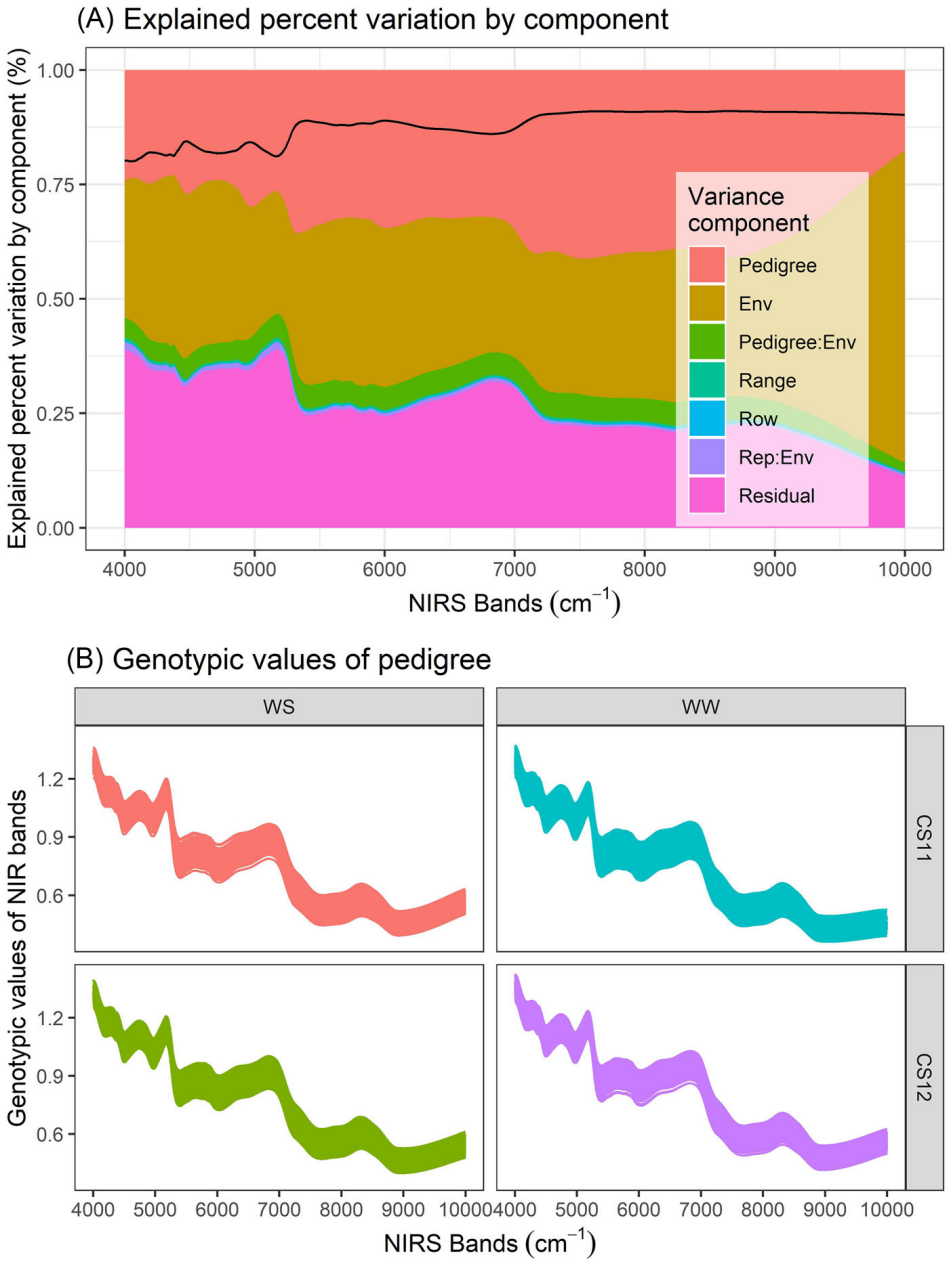
(A) Explained percent variation across all 3112 near‐infrared spectroscopy (NIRS) bands in each of the four environments as computed by Equation (1), where Yjklmn=μ+Pedigreej+Envk+Env×Pedigreejk+Rangel+Rowm+Env×Repnk+εjklmn. (A) is displayed as a proportional stacked area chart. The solid black line denotes repeatability values as they change across the bands (Equation 2). (B) Genotypic values of all NIRS bands are displayed for each environment as calculated in Equation (1). CS, College Station; WS, water‐stressed; WW, well‐watered.

The results of ANOVA using Equation (1) across all 3112 NIRS wavelength bands revealed variability in percentages of explained variation in different regions of the spectrum, with pedigree accounting for as much as 41.2% at ∼7532 cm^−1^ and as little as 17.6% at ∼9997 cm^−1^ (Figure 2A). On average, pedigree variance explained a comparable percentage of experimental variation across the spectrum for GY and KW (33.0% vs. 35.1%, respectively), while the third‐largest component was environmental variation (25.6%). The interaction between pedigree and environment explained <5% of variation. Spatial effects such as range and row accounted for <5% and 3%, respectively, while the interaction between replication and environment on average accounted for 7.70%. Repeatability, as computed by Equation (2), ranged between 0.800 and 0.910 (given by the solid black line in Figure 2A). The highest overall values for repeatability were observed between ∼7100 and 10,000 cm^−1^. Phenotypic values of NIRS bands over the entire spectrum followed similar trends in each environment (Figure 2B).

### 3.3 Assessment of prediction ability

**FIGURE 3:**
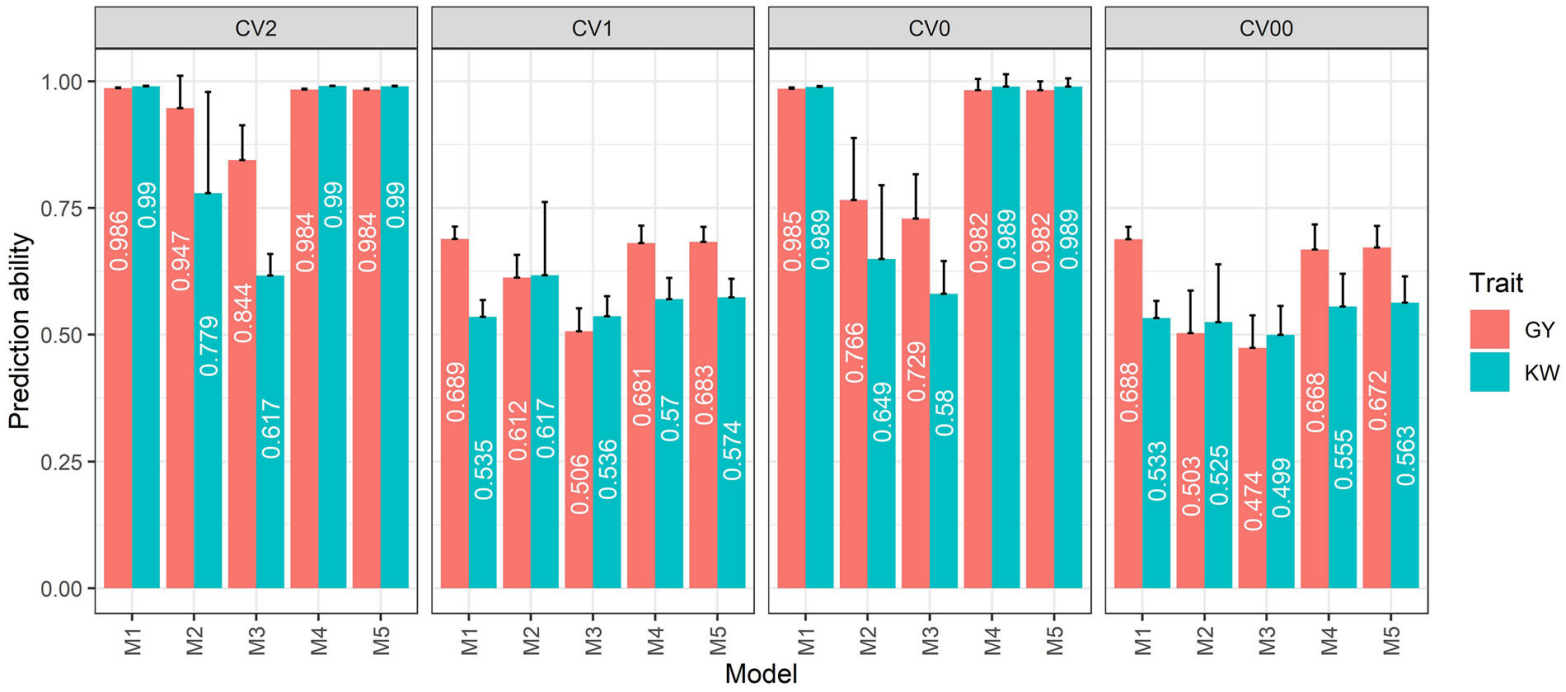
Prediction abilities of each across‐environment prediction model for grain yield (GY) and 500‐kernel weight (KW) are displayed within each cross‐validation scheme. Red indicates GY, and blue indicates KW. Model notation is described in detail in Table 1. Error bars denote standard deviations (only the top bracket is shown). M1: genomic (g), environment (E), plus their interaction (gE); M2: phenomic (q), environment (E), plus their interaction (qE); M3: phenomic with only near‐infrared spectroscopy (NIRS) bands nominated by lasso (216 bands) (qs), environment (E), plus their interaction (qsE); M4: combination of M1 and M2; M5: combination of M1 and M3. CV, cross‐validation.

Average prediction abilities for all models grouped by CV and trait (in order of GY and KW) were 0.949 ± 0.028 and 0.873 ± 0.049 for CV2, 0.634 ± 0.036 and 0.566 ± 0.059 for CV1, 0.889 ± 0.050 and 0.839 ± 0.051 for CV0, and lastly, 0.601 ± 0.053 and 0.535 ± 0.064 for CV00 (Figure 3). Within CV2 and CV0, in which tested hybrids are predicted in characterized and uncharacterized environments, respectively, genomic (M1), combined genomic plus phenomic (M4), and genomic plus subset phenomic (M5) models performed nearly identically, each above ∼0.98.

For GY prediction of untested genotypes in characterized environments (CV1), genomic prediction (M1) had the highest accuracy (0.689 ± 0.024), with PP trailing by 12.5% at 0.612 ± 0.045. The model incorporating the phenomic relationship matrix derived from a subset of 216 lasso nominated NIRS bands achieved a prediction ability of 0.506 ± 0.045. Multi‐kernel methods (M4 and M5) did not outperform M1. Prediction of GY in CV00, where phenotypic values of untested genotypes are predicted in uncharacterized environments, was again best performed by M1 (0.688 ± 0.024).

Prediction of KW in CV1 was best performed by M2, though this model had a higher standard deviation of prediction than M1 (0.617 ± 0.150 vs. 0.535 ± 0.034, respectively). Neither multi‐kernel model was able to surpass the performance of M2, though M4 and M5 had standard deviations of prediction that were 71.1% and 74.7% lower, respectively. In CV00, KW was predicted best by the multi‐kernel genomic plus subset phenomic model (M5, 0.563 ± 0.052).

### 3.4 Correlations of NIRS bands with GY, KW, and all pairwise bands

**FIGURE 4:**
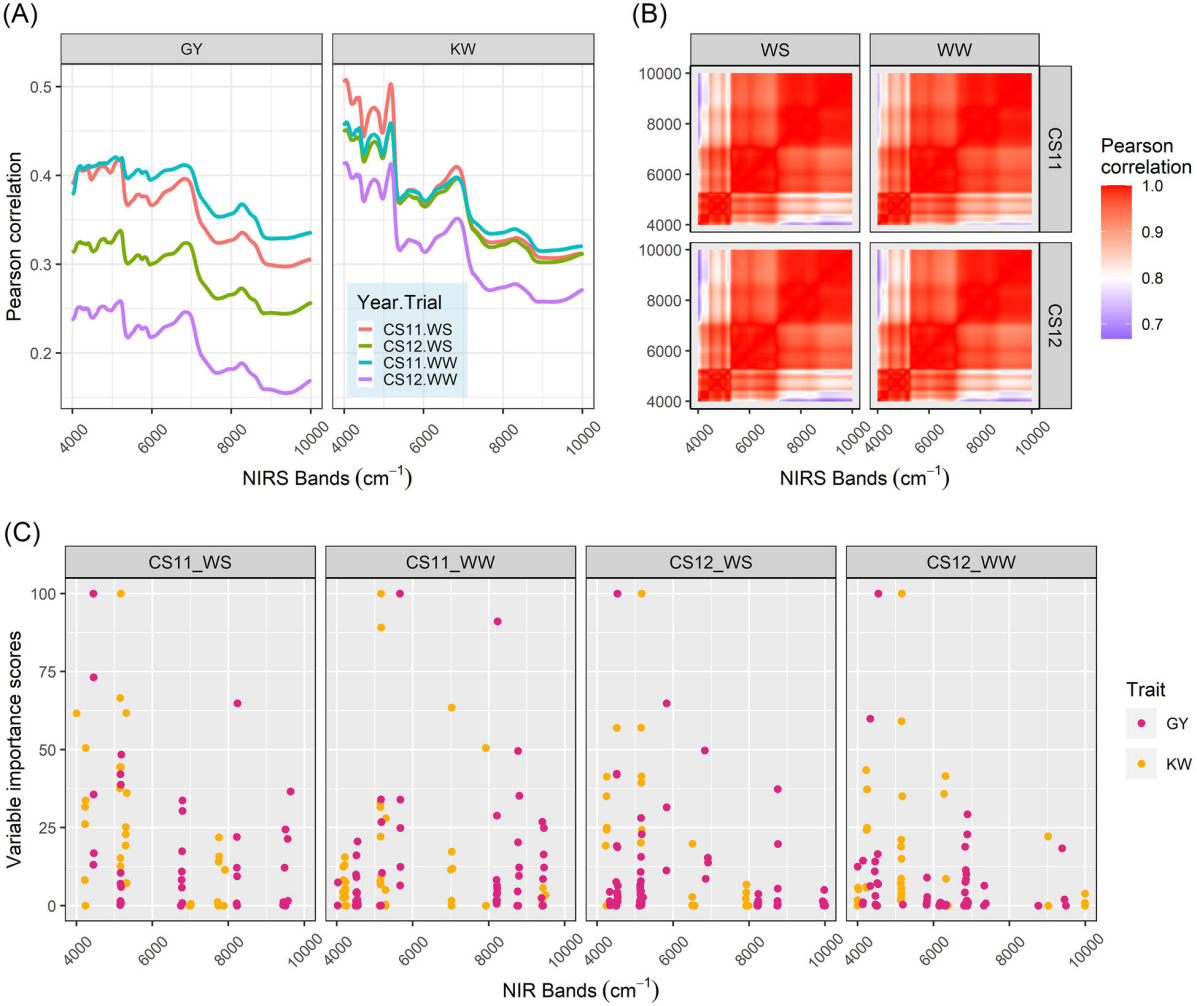
(**A**) The correlations of grain yield (GY) and 500‐kernel weight (KW) with each near‐infrared spectroscopy (NIRS) band. Colors denote each of the four environments. (B) The correlations of the 3112 NIRS bands between each other separated by environment. (C) Variable importance scores calculated by the lasso regression are reported for GY and KW in each environment. Bands with high variable importance are displayed as points with values closer to 100. Lasso nominated 216 and 133 NIRS bands for GY and KW, respectively. Subset phenomic prediction was performed with bands nominated for GY, as these were advantages over subset prediction using KW alone or GY and KW combined. CS, College Station; NIR, Near‐infrared reflectance; NIRS, near‐infrared spectroscopy; WS, water‐stressed; WW, well‐watered.

Similar patterns of correlation between GY and KW with the NIRS bands were observed in each of the four environments, however, spectra from CS11_WS had the highest average correlation for GY (averaging 0.378 across the entire spectrum), while CS12_WW was markedly lower at 0.207 (Figure 4A). Correlations between NIRS bands and KW across the four environments were more tightly grouped (Figure 4A). For both GY and KW, correlation trends decreased as the wavelengths increased (Figure 4A). Correlations of the 3112 NIRS bands with each other revealed that every pairwise correlation was above 0.66 (Figure 4B), prompting dissection of the NIRS data set using lasso (Figure 4C). Three main clusters of NIRS bands were visible and consistent across four environments (e.g., cluster 1: ∼4000–5000 cm^−1^; cluster 2: ∼5000–7000 cm^−1^; and cluster 3: ∼7000–10,000 cm^−1^) (Figure 4B). Certain NIRS bands displayed consistency across environments and both traits, such as in the range of ∼5000–∼5200 cm^−1^ (Figure 4C).

## 4 DISCUSSION

Improvements in historical crop productivity are largely attributable to traditional plant breeding methods (Evenson & Gollin, 2003) and improved agronomic practices (Rizzo et al., 2022). Recent improvements of classical techniques are due in part to the integration of genomic selection into breeding paradigms. Phenomic prediction and selection approaches may further improve this paradigm, both through increased measurement quantity leading to more accurate genetic value estimations (Lane & Murray, 2021) and through better understanding and modeling of genetic relationships based on how genotypes interact with their environments. Given the novelty of NIRS‐based PP approaches, this technology has yet to be integrated into breeding programs at scale. The present study builds on the body of knowledge demonstrated by other studies regarding the potential value of NIRS to predict agronomic field performance (Brault et al., 2022; Rincent et al., 2018; Weiß et al., 2022), however, it highlights limitations of the technique when applied in a high‐throughput manner to scans of whole unground maize grain. Variance component estimation obtained from Equation (1) along all 3112 NIRS bands demonstrated that pedigree variance accounted for between 33.0% and 35.1% of variation with moderate to high repeatability (Figure 2A), indicating that reflectance values obtained by NIRS have the potential to capture effects related to the unique genetic backgrounds of the germplasm that were scanned.

Here, we used whole grain intact samples as opposed to finely ground, homogenized samples. This method affords much greater throughput and practicality but likely lowers prediction ability due to the shallow depth of light penetration into the sample, sample heterogeneity, and inconsistent light scattering. This may have been a critical limitation in the present study and offers a likely explanation why genomic prediction (M1) was better in some scenarios (GY prediction in CV00 and CV1) than PP with all NIRS bands (M2) (Figure 3). Spielbauer et al. (2009) hypothesized that the larger and asymmetric nature of grain can produce differing NIRS reflectance values for whole grain depending on whether the germinal or abgerminal side is facing the instrument (Heffner et al., 2011; Orman & Schumann, 1992; Weinstock et al., 2006). A potentially insightful follow‐up to this study might be to scan finely ground kernel samples with NIRS to see if the scanning methodology improves the predictive ability of the PP models (M2 and/or M3). Alternatively, to avoid the laborious process of grinding samples, hyperspectral imaging via novel methods developed by Varela et al. (2022) may allow researchers to obtain accurate estimations of kernel composition traits nondestructively, in addition to obtaining morphometric features such as width and length of the kernels. An additional limitation to the present study's findings on the use of PP includes the limited effects of G × E for GY and KW, as the interaction effect between pedigree and environment accounted for only 4.04% and 3.57% for GY and KW, respectively (Figure 1A). This phenomenon was also observable in the ANOVA results of each NIRS band, with the average percentage explained by the interaction between pedigree and environment only reaching 4.71%. Additional G × E may have increased the value of PP when compared with genomic prediction.

### 4.1 CV1 and CV00 prediction results

For plant breeding programs, CV1 (prediction of untested genotypes in tested environments) and CV00 (prediction of untested genotypes in untested environments) are the most realistic and applicable scenarios, as it is desirable to predict the performance of new genotypes in either familiar or new trial locations. For GY in CV1 and CV00, genomic prediction (M1) performed better than PP alone (M2) and similarly to genomic plus PP (M4), indicating that a trait measured with relatively high repeatability in diverse environments in this study could sufficiently be predicted by genomic data while phenomic data added little value (Figure 3). The latter point contrasts with the findings of Rincent et al. (2018), in which NIR spectra from wheat leaf tissue collected in one environment generated an estimated covariance matrix capable of accurate yield predictions in a weakly correlated separate environment (located ∼500 km away). Future studies are warranted to determine whether NIRS‐based PP is suitable as environments become more disparate. However, in CV1, KW was best predicted by M2 (which used the entire NIR spectrum), even surpassing combined genomic plus PP (M4). Prediction of KW by M2 in CV00 was on par with M1, differing by only 1% accuracy. The mixed performance of M2 versus M1 indicates that, despite not always being able to surpass genomic prediction, NIRS‐based PP can still perform comparably and may add value to research programs as a nondestructive mechanism of spectral measurement.

Lane et al. (2020) demonstrated the utility of using a functional regression model to predict untested hybrids in two out of four environments (predicting 20% of hybrids untested in two environments but tested in the remaining two), reporting a prediction ability of 0.87 when supplying the model with phenotypic data from WW environments while withholding information from WS. The NIRS BLUP model in the same prediction scenario fared worse, reporting a prediction ability of 0.07. The methods of this study differed from Lane et al. (2020) (namely, from the use of functional and partial least squares [PLS] regression) to test the prediction ability of NIR spectra when treated in a similar manner to genomic marker relationship matrices. The variable selection imposed by PLS in Lane et al. (2020) may have handled the autocorrelation observed when employing the entire spectrum for prediction, and to test this, lasso was used to filter through autocorrelated bands before creating a phenomic relationship matrix based only on important NIRS wavelengths (M3). In CV1 and CV00, M3 performed similarly to M2, indicating that the majority of the NIR spectrum was redundant. Combined genomic and phenomic (M4) and combined genomic and subsetted phenomic (M5) prediction models did not surpass genomic prediction alone for GY, suggesting that at least in this study's germplasm, genomic and phenomic data sets captured similar yield‐related features and did not lead to synergistic effects when data modalities were combined. With KW, however, modest improvements in prediction ability were observed with M4 and M5 performance in CV1 and CV00 compared with phenomic or genomic prediction alone (M1/M2/M3), indicating a trait dependency for the use cases of NIRS relationship matrices in predicting agronomic traits.

### 4.2 Applicability of genomic and phenomic data for plant breeding

As an alternative or supplement to genomic prediction, PP uses spectral signals instead of molecular markers. As reflectance spectra are representative of tissue biochemical properties, which are genetically controlled, relationship matrices derived from plant spectra can capture genetic signals (Brault et al., 2022) but also likely environmental and genome × environmental signals. The advent of affordable remote sensing technologies has ushered phenomics into an era of low‐cost and higher throughput data collection, in which spectral data sets (RGB, multispectral, or hyperspectral) enable calculation of vegetation indices that can parameterize predictive models that inform selection decisions (Adak et al., 2021; Galán et al., 2020; Hernandez et al., 2015; Montesinos‐López et al., 2017; Rutkoski et al., 2016). Because these approaches and demonstrations are new compared to more extensively investigated genomic approaches, case studies are needed to build knowledge sufficient to improve phenomic methods. Brault et al. (2022) demonstrated that similarities existed between NIRS and genomic relationship matrices across tissues or years in *Vitis vinifera* (grapevine). These authors, like many others reported to date, found that despite modest genetic variance estimated from NIRS, PP was comparable to that of GP.

In this study, genomic prediction (M1) frequently surpassed the predictive ability of phenomic (M2), subset phenomic (M3), or combined genomic/PP (M4/M5), indicating continued merit in sparse prediction for multi‐environment trials. While there were scenarios (KW in CV1 and CV00) in which inclusion of NIR spectra with genomic relationship matrices improved prediction abilities, scans of intact maize kernels may not surpass the performance of genomic prediction due to the nature of asymmetric nutrient deposition during grain filling as discussed previously (Figure 3). However, PP was frequently able to achieve prediction abilities on par with genomic prediction. In this study, samples were scanned nondestructively and there were no laboratory consumables besides electricity for the scanner required to obtain the spectral data. As such, NIRS may (after an initial investment in the spectrometer) serve as an economical and rapid‐turnaround alternative to genomic data while potentially providing simultaneous estimates of kernel composition traits such as phenolic antioxidants (Meng et al., 2015).

A further advantage of phenomic measurement technologies such as NIRS is the ability to record environment‐specific and genetic by environment‐specific metrics with the intent of capturing phenotypic plasticity of the same genotypes grown in disparate environments (Zhu et al., 2021a). However, this advantage is less pronounced with NIRS, as scans must be conducted after the germplasm being studied has been harvested. Given the sensitivity of genomic prediction and selection to training/testing set relatedness (Brauner et al., 2020; Olatoye et al., 2020; Riedelsheimer et al., 2012; Weiß et al., 2022; Zhu et al., 2021b), PP has shown merit in predicting performance of diverse breeding material with varying degrees of relatedness (Weiß et al., 2022). Zhu et al. (2021b) and Brauner et al. (2020) reported decreasing genomic prediction ability as the training set composition moved from full‐sib to half‐sib to unrelated families in *Glycine max* (L.) Merr. and maize, respectively. The diversity of the hybrid panel in the present study may also have played a role in the ability for models to predict across environments and serves as a key distinction between the elite wheat varieties and association mapping panel of poplar reported by Rincent et al. (2018). Though NIRS‐based PP alone underperformed genomic prediction in this study for GY, there may still be advantages to phenomic approaches for plant breeders owing to its rapid turnaround time and fixed costs versus the consumable nature of preparing samples for genotyping. This will be increasingly true where NIRS pipelines are more automated, including NIRS integrated on combine harvesters, though these too will likely suffer from uneven nutrient deposition if applied to maize kernels. As a result, phenomic approaches may enable increased selection accuracy with existing population sizes or selection intensity if they allow more lines and hybrids to be screened. Furthermore, NIRS phenomic‐based approaches could provide additional value in correcting errors in combine yield data or predicting other unmeasurable traits (e.g., flowering time) not directly estimable from either DNA or conventional grain composition measurements.

It is important to note that using NIRS on hybrid F_1_ seed before planting would likely be inappropriate in the case of hybrid maize, where the kernel would represent the inbred mother plant and not the genetic potential of the hybrid that will emerge from the seed. In contrast, using the harvested grain, assuming no xenia effect (which is reasonable in this study, Bulant et al. [2000]), will result in estimations of yield from the plants this grain was selected from. In the case of mechanically (e.g., combine) harvested yield trials, this is unlikely to provide any substantial speed advantages regarding mid‐growing season selection decisions, but the enhancement in prediction ability afforded by NIRS data could reduce the amount of screening required in subsequent seasons because genomic/PP results can be implemented for either positive or negative selection, especially in untested environments. Though this study examined the predictive ability of spectra obtained from harvested hybrid F_2_ grain, future investigation of predicting testcross performance from NIRS data obtained from inbred grain is warranted and would have direct relevance for breeding programs. This concept has been explored for genomic prediction of hybrid maize GY using doubled haploid lines (Albrecht et al., 2011) and hybrid sorghum yield [*Sorghum bicolor* (L.) Moench] using elite inbred lines (Fonseca et al., 2021), but has received little attention from the phenomics research community. Demonstration of comparable prediction accuracies for predicting hybrid performance using phenomic data acquired for inbreds (i.e., NIRS, vegetation indices from unoccupied aerial system [UAS]) could provide breeders with low‐cost alternatives to genotyping inbred parents.

Prior studies have demonstrated an array of statistical treatments for NIRS in PS since most models applicable to genomic selection can be adapted to phenomic selection (PS) (Robert, Brault et al., 2022). Functional regression, in which observations are functions outlined on a continuous domain, has also been implemented in PS for yield prediction, leading to greater parsimony among regression models and higher computational efficiency (Lane et al., 2020; Montesinos‐López et al., 2018, 2017; Morris, 2015). Variable selection techniques such as lasso and BayesB can reduce multicollinearity and high dimensionality characteristic of NIRS data, where large numbers of wavelengths supersede the number of observations (Lane et al., 2020; Robert, Brault et al., 2022). Variable selection in PS can improve interpretability of findings, as certain predictors (in the case of this research, NIRS bands) could show higher heritability than others (Ferragina et al., 2015; Galán et al., 2020). However, Aguate et al. (2017) and Galán et al. (2021) both reported losses in prediction accuracies when less than the full wavelength spectrum (hyperspectral data in these cases) was used, and minor decreases in prediction ability in M3 versus M2 were also observed in the present study (Figure 3). Assessment of variable importance in this study among NIRS bands for GY (Figure 4C) via the lasso regression highlighted wavelengths such as ∼5000–5200 cm^−1^ and indicated a large portion of the spectrum was autocorrelated. As noted by Zhu et al. (2021a), determining a smaller group of predictive bands creates the potential for production of lower‐cost spectrometers that omit non‐predictive or repetitive bands.

### 4.3 Future directions

The push to explore the predictive nature of agricultural phenomic data has led to the adoption of UASs (or drones) to capture temporal, also called longitudinal, phenomic data at scale, which enables the elucidation of genotypic variation across a growing season (Adak et al., 2021) and potentially well before harvest. In contrast, genomic information and NIRS of grain samples represent single measurements, which is a primary limitation of the present investigation. Future studies are warranted that take advantage of NIRS, RGB, multispectral, and even hyperspectral relationship matrices in attempting to perform prediction of agronomically relevant traits using novel statistical methods to handle large numbers of predictors throughout growth.

## 5 CONCLUSIONS

Overall, genomic‐only prediction was more suited to across‐environment prediction of GY of untested genotypes in characterized and uncharacterized environments, while combined genomic/PP performed best for prediction of 500‐kernel weight. NIR spectra were able to delineate between pedigree, environment, spatial, and interactions between pedigree and environment, although these results indicated that high‐throughput scans of intact maize kernels may not be able to surpass genomic prediction when used in a relationship matrix prediction context. However, the nondestructive nature of NIRS whole‐kernel scans, coupled with its comparable prediction results to genomic prediction, indicates potential value within research programs for selection. Further study of integrating phenomic and genomic data is necessary to enable more accurate and efficient prediction models, reduced need for field‐based phenotyping, and an accelerated breeding process. Phenomic relationship matrices using NIRS bands nominated by lasso enabled comparable prediction abilities to relationship matrices based on the entire NIR spectrum, indicating that, as with genomic markers, high degrees of autocorrelation were present in the phenomic data set. Future studies of combining NIRS and genomic data have the potential to overcome some of the limitations of each individual technique, providing a more comprehensive understanding of the genetic and physiological factors that contribute to both agronomically important and research‐specific traits in maize such as GY and KW, which is especially fitting for breeding programs targeting genetic gains and developing locally adapted germplasm.

## AUTHOR CONTRIBUTIONS

**Aaron J. DeSalvio**: Conceptualization; data curation; formal analysis; investigation; methodology; visualization; writing—original draft; writing—review and editing. **Alper Adak**: Conceptualization; data curation; formal analysis; investigation; methodology; visualization; writing—original draft; writing—review and editing. **Seth C. Murray**: Conceptualization; data curation; funding acquisition; project administration; supervision; writing—review and editing. **Diego Jarquin**: Formal analysis; methodology; writing—review and editing. **Noah D. Winans**: Formal analysis; methodology; writing—review and editing. **Daniel Crozier**: Formal analysis; methodology; writing—review and editing. **William L. Rooney**: Conceptualization; methodology; writing—review and editing.

## CONFLICT OF INTEREST STATEMENT

The authors declare no conflicts of interests.

## Supporting information

Supporting Information

Supporting Information

**Supp. Fig. 1** provides a visual representation of the numbers of hybrids grown in each of the four environments, outlining which hybrids are shared and which are unique to a given environment. **Supp. Fig. 2** details a visual representation of the architecture of each cross‐validation scenario, highlighting which hybrids were used for training and testing each of the models.

## Data Availability

An annotated script of the R code used in this research can be accessed via GitHub (DeSalvio, 2023) [https://github.com/ajdesalvio/Maize‐NIRS‐GBS.git]. All files needed to reproduce the results are provided in the GitHub repository for users to access.
