# Supplementary material for: Near‐infrared reflectance spectroscopy phenomic prediction can perform similarly to genomic prediction of maize agronomic traits across environments
Source: Plant Genome. 2024 May 7;17(2):e20454. doi: 10.1002/tpg2.20454 (PMC12807438; doi:10.1002/tpg2.20454)
Supplement: Supplementary file 1 [file TPG2-17-e20454-s002.docx]

**Supplementary Figure 1**. 146 maize hybrids were common to all four environments. In total, CS11_WS contained 196 hybrids, CS11_WW contained 263 hybrids, CS12_WS contained 310 hybrids, and CS12_WW contained 213 hybrids.

**Supplementary Figure 2**. All numbers within small blue, orange/tan, and gray boxes represent quantities of hybrids. Blue boxes denote approximately equally sized folds of common hybrids (146 total common hybrids found in each environment). Orange/tan boxes indicate the unique hybrids grown in each location, with quantities varying by location. The structures of the training data sets are pictured in the top two sections that are titled “Training”. In the sections titled “Evaluation”, the models were assigned accuracy values based on the correlations between actual and predicted values of the hybrids indicated by the blue and/or tan boxes. Blue indicates the phenotypic records for hybrids that were present in the training data set while gray indicates the records were omitted.
